# Photonic Floquet topological insulators in a fractal lattice

**DOI:** 10.1038/s41377-020-00354-z

**Published:** 2020-07-20

**Authors:** Zhaoju Yang, Eran Lustig, Yaakov Lumer, Mordechai Segev

**Affiliations:** Physics Department and Solid State Institute, Technion–Israel Institute of Technology, Haifa, 32000 Israel

**Keywords:** Optical physics, Optical physics

## Abstract

We present Floquet fractal topological insulators: photonic topological insulators in a fractal-dimensional lattice consisting of helical waveguides. The helical modulation induces an artificial gauge field and leads to a trivial-to-topological phase transition. The quasi-energy spectrum shows the existence of topological edge states corresponding to real-space Chern number 1. We study the propagation of light along the outer edges of the fractal lattice and find that wavepackets move along the edges without penetrating into the bulk or backscattering even in the presence of disorder. In a similar vein, we find that the inner edges of the fractal lattice also exhibit robust transport when the fractal is of sufficiently high generation. Finally, we find topological edge states that span the circumference of a hybrid half-fractal, half-honeycomb lattice, passing from the edge of the honeycomb lattice to the edge of the fractal structure virtually without scattering, despite the transition from two dimensions to a fractal dimension. Our system offers a realizable experimental platform to study topological fractals and provides new directions for exploring topological physics.

## Topological photonics: fractal lattices

Photonic topological insulators are currently a subject of great interest because they support edge states that can propagate without being affected by defects and disorder. All topological insulators discovered thus far have a bulk surrounded by edges. Now, Zhaoju Yang and coworkers from Technion in Israel, found theoretically that photonic topological insulators can also exist in fractal lattices, comprising only edges—with no bulk at all. They studied fractal lattices structured as Sierpinski gasket composed of an array of evanescently coupled helical waveguides. Despite the lack periodicity in such structures, tight-binding simulations and quasienergy analysis predict the existence of topological edge states, residing either on outer or on inner edges, exhibiting a Chern number of 1 and displaying scattering-free propagation. The fractal symmetries of such lattices are found to be crucial for the existence of the topological properties. Such fractal lattices could be fabricated by femtosecond laser writing technology.

## Introduction

Topological insulators are a new phase of matter characterized by an insulating bulk and perfectly conductive edges^1,2^. They have been at the forefront of condensed matter physics for the past decade and more recently inspired the emergence of topological phases in many classical-wave systems^3–5^, such as microwaves^6–8^, photonics^3,9–16^, acoustics^4^, and more. Photonics specifically has become the cutting-edge platform for exploring all kinds of topological phases ranging from the quantum spin Hall effect^12^, Floquet topological insulators^13^, topological crystalline insulator^16^, and valley Hall effect^17,18^; all the way to topological systems that lack periodicity, such as topological quasicrystals^19^ and even topological Anderson insulators, in which the topology is induced by disorder^20^. Thus far, all studies of topological insulators have explored systems in integer dimensions (physically, 2D or 3D) with a well-defined bulk and edges. However, the physical dimensions do not always define the dimensions in which a system evolves: some structures have a non-integer (fractal) dimension, despite being in a 2D or 3D realm. The existence of systems with fractal dimensions raises a series of fascinating questions in the context of topological physics. For example, is it possible to realize topological edge states in fractal dimensions? Moreover, fractal structures tend to include holes, so can topological edge states be found around every hole in the system or only in the external boundary? Intuitively, one might think that there are no topological edge states because our fractal lattices often contain no bulk at all, hence one cannot rely on the bulk-edge correspondence^21,22^ to predict topological edge states in fractal lattices. This raises a deeper question: is there bulk-edge correspondence when the fractal structure is actually made up of holes within the bulk?

Here, we investigate the photonic Floquet topological phase in a periodically driven fractal lattice. This lattice relies on a fractal photonic crystal [the Sierpinski gasket (SG)] consisting of evanescently coupled helical waveguides, which can be realized by femtosecond-laser-writing technology^23^. We calculate the topological Floquet spectrum and show the existence of topological edge states corresponding to real-space Chern number 1^24,25^, which can be controlled by periodic driving. We explore the dynamics of the edge states and their robustness in simulations in the fractal SG lattice and find that wavepackets made up of topological edge states propagate along the outer edge without penetration into the bulk and without backscattering even in the presence of disorder and sharp corners. Likewise, the topological edge states associated with inner edges in the fractal lattice exhibit robust transport whenever the inner edge includes a large enough area. These results imply that fractal structures can act as topological insulators, despite the lack of periodicity and the structures being made up mostly of holes. Subsequently, we study transport in a hybrid lattice combining the fractal lattice with a honeycomb lattice and find that topological edge states can pass from the honeycomb lattice into the edge of the fractal lattice and vice versa, where they exhibit topologically protected transport. This observation further demonstrate that the edge states in the fractal lattice directly correspond to the same Chern number as that of a honeycomb lattice driven by the same periodic modulation. Finally, it is possible to obtain similar results with other fractal platforms: the Sierpinski carpet under an aperiodic arrangement, and we conjecture that the 3D realizations of both the SG and the Sierpinski carpet also give rise to topological edge states and, likewise, the Cantor cubes and Cantor dust. Hence, our results suggest a wealth of new kinds of topological systems and new applications, such as using topological robustness combined with the enhanced sensitivity of fractal systems for sensing and, in non-Hermitian settings, topological insulator lasers^26–28^ in fractal dimensions.

## Results

**Fig. 1:**
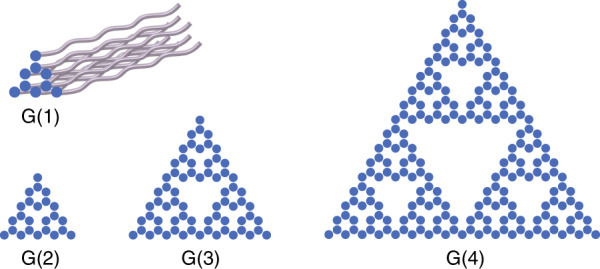
Iterative generations of the Sierpinski gasket (SG). The first generation G(1) of the SG has nine blue sites. G(*n*) has three copies of G(*n*−1), sharing three corner sites. The fractal lattice of helical waveguides is generation G(4) with a total of 204 sites. Each blue site marks the position of a helical waveguide. The helicity of the waveguides introduces an artificial vector potential ${\boldsymbol{A}}\left( z \right) = A_0\left[ {\sin \left( {{\mathrm{\Omega }}z} \right), - \cos \left( {{\mathrm{\Omega }}z} \right),0} \right]$. For presentation simplicity, we draw only the three-dimensional schematic of a G(1) lattice of helical waveguides

Our starting point is the SG with a Hausdorff dimension $d_f = {\mathrm{ln}}\left( 3 \right)/{\mathrm{ln}}\left( 2 \right) = 1.585$. Consider a photonic lattice of evanescently coupled helical waveguides, similar to ref. ^13^. Figure 1 shows the iterative generations of the SG. As can be seen, the first generation G(1) of the SG has nine blue circles, which indicate the positions of the helical waveguides. Generation G(2) consists of three copies of G(1), sharing three vertices. Accordingly, the G(2) waveguide lattice has 24 waveguides organized as the second generation of the SG. Similarly, G(*n*) has three copies of G(*n* − 1), sharing three corner sites. Hereafter, we focus on fractal lattices of generations G(4) and G(5), and we conjecture that the conclusions we draw from this study hold for the SG lattice in any generation. Examining Fig. 1 reveals that all sites in the SG fractal lattice are on the boundaries, and there is not even a single site that does not reside on a boundary—external or internal. Finally, as in ref. ^13^, this lattice consists of helical waveguides, which is equivalent to a periodically driven potential that introduces an artificial gauge field ${\boldsymbol{A}}$.

The equation governing the diffraction of light in this fractal photonic lattice under the tight-binding approximation^13^ can be written as

1 $$i\partial _z\psi _n = c_0\mathop {\sum }\limits_{<{m}>} e^{i{\boldsymbol{A}}\left( z \right) \cdot {\boldsymbol{r}}_{m,n}}\psi _m$$

where *z* is the optical axis, $\psi _n$ is the amplitude of the electric field in the *n*th waveguide, $c_0$ is the coupling strength, ${\boldsymbol{r}}_{m,n}$ is the displacement vector pointing from waveguide *m* to waveguide *n*, ${\boldsymbol{A}}\left( z \right) = A_0\left[ {\sin \left( {{\mathrm{\Omega }}z} \right), - \cos \left( {{\mathrm{\Omega }}z} \right),0} \right]$ is the artificial vector potential induced by the helicity of the waveguides with amplitude $A_0 = kR{\mathrm{\Omega }}$, in which $k$ is the wavenumber of the light in the medium, $R$ is the radius of the helix, ${\mathrm{\Omega }}$ is the longitudinal frequency of the helix corresponding to periodicity $L = 2\pi /{\mathrm{\Omega }}$, and m indicates that the summation is taken over all the nearest waveguides to waveguide *n*. The light evolution in the system is described by the paraxial wave equation, which is mathematically equivalent to the Schrödinger equation, with the *z*-axis playing the role of time. Equation (1) is derived by applying the tight-binding approximation to the paraxial wave equation.

**Fig. 2:**
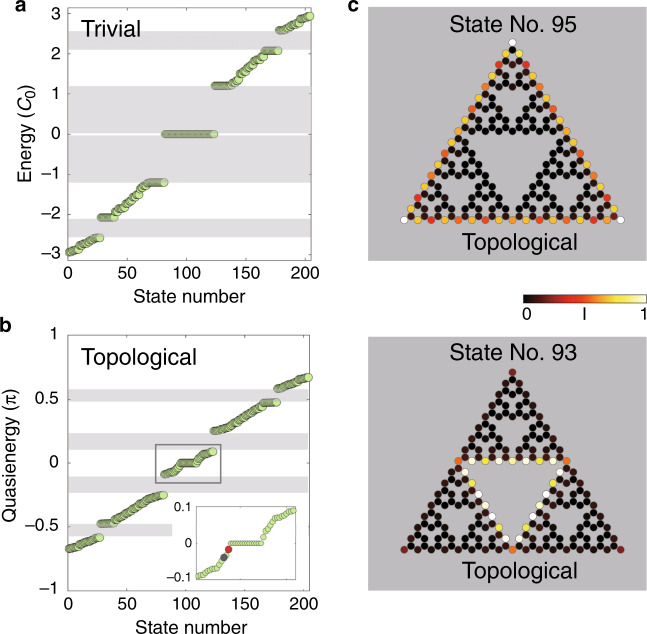
Eigen-states of the fractal lattice. **a** Energy spectrum with ${\boldsymbol{A}}\left( z \right) = 0$ (straight waveguides). The spectrum of this non-topological system displays a large central gap (large gray region), with a flat band in the mid-gap made up of immobile degenerate states. **b** Quasi-energy spectrum with ${\boldsymbol{A}}\left( z \right) \ne 0$ and $A_0 = kR{\mathrm{\Omega }}$. The inset shows an enlarged view of the center box. The shaded regions mark quasi-gaps: regions within which there are no eigen-states. In this topological fractal system, the edge states from either side of the flat band evolve into nondegenerate unidirectional edge states. **c** Field intensity patterns of two eigen-states localized at the external and internal edges of the fractal lattice (states 93 and 95, gray and red dots, respective√ly). The color bar indicates the intensity (normalized to the peak intensity in each state). The parameters used are the ambient refractive index $n_0 = 1.45$, coupling strength $c_0 = 1.9\,{\rm{cm}}^{ - 1}$, wavelength $\lambda = 0.633$ µm, helix radius $R = 10$ µm, longitudinal frequency of the helix ${\mathrm{\Omega }} = 2\pi \,{\rm{cm}}^{ - 1}$, and lattice constant $a = 14\sqrt 3$ µm

The eigen-values and eigen-states can be obtained by diagonalizing the unitary evolution operator for one period^29^. The results of the quasi-energy spectrum $\beta$ (which in a photonic lattice are the deviation of the propagation constant from the wavenumber in the medium^13^) in the fractal SG systems are shown in Fig. 2a, with ${\boldsymbol{A}}\left( z \right) = 0$ corresponding to the straight waveguides, and in Fig. 2b, with ${\boldsymbol{A}}\left( z \right)\,\ne\, 0$ corresponding to the helical waveguides. The spectrum for our G(4) fractal lattice is organized into five bunches (“bands”) separated by gaps (gray shaded regions in Fig. 2a, b). The spectrum of the non-topological system (Fig. 2a) shows a large central gap, with a flat “band” in the mid-gap. These states are immobile and degenerate (they all have the same energy), as expected from a non-topological system. On the other hand, for the driven system (the helical waveguides), as shown in Fig. 2b, the edge states from the central flat band evolve into nondegenerate unidirectional edge states. Figure 2c shows the field intensities of the actual wavefunctions of these eigen-states, specifically states 93 and 95 (out of 204 eigen-states), with quasi-energies of −0.040 and −0.018, respectively. These states are localized at the exterior (state number 95) and the interior (state number 93) edges. As we show below, these states behave as topological edge states, exhibiting Chern number 1 and topologically protected transport. Note that in other bunches with quasi-energies below −0.2 or above 0.2, the eigenstates are bulk states.

**Fig. 3:**
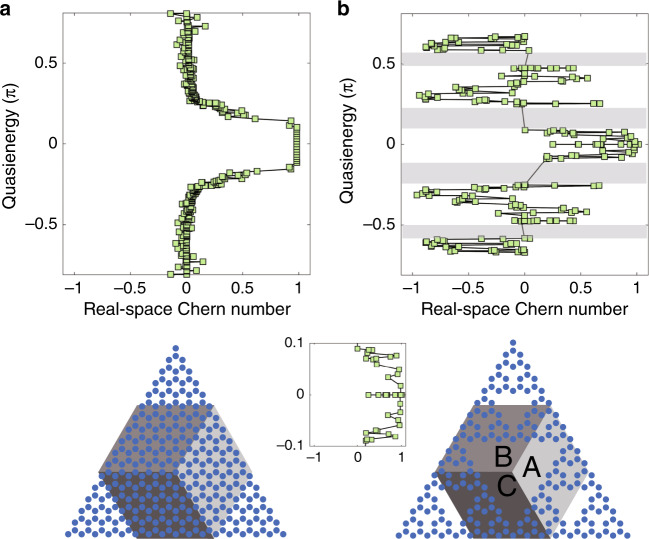
The real-space Chern number as a function of the quasi-energy for the honeycomb (a) and the fractal (b) lattices with the same nonzero artificial gauge field. The lower panels present the honeycomb and the fractal lattices, which are both triangular-shaped. When calculating the real-space Chern number, the hexagons are divided into three regions with different shades of gray, each enclosing many waveguides. The center inset shows an enlarged view of the quasi-energy range of the fractal lattice from −0.1 to 0.1. The parameters of the lattices are the same as in Fig. 2

To verify that the edge states we have found [the non-degenerate unidirectional states in the rectangle of Fig. 2b, two of which are shown in Fig. 2c] are indeed topological, we need to characterize our system through its Chern number. Since fractal lattices are nonperiodic, we calculate the real-space Chern number^24,25^. Heuristically, the real-space Chern number “measures” the chirality of states at a specific quasi-energy, and in periodic systems, it yields the same integer number as the “standard” Chern number (defined on the momentum space)^24,25^. The definition of the real-space Chern number is

2 $$C = 12\pi i\mathop {\sum }\limits_{j \in A} \mathop {\sum }\limits_{k \in B} \mathop {\sum }\limits_{l \in C} \left( {P_{jk}P_{kl}P_{lj} - P_{jl}P_{lk}P_{kj}} \right)$$

where $j,k,l$ are the lattice site indices within three different neighboring regions A–C [as drawn in the inset of Fig. 3b, arranged anti-clockwise], $P_{jk} = \langle{j{\mathrm{|}}P{\mathrm{|}}k}\rangle$ and the projector operator $P$ projects onto a given state of a specific quasi-energy (a state with quasi-energy playing the role of the Fermi level). The results are shown in Fig. 3a, b. We calculate the real-space Chern number for our fractal lattice and, for a direct comparison, also for a honeycomb lattice, with both being driven by the same periodic modulation (manifested here as the helicity of the waveguides). The lower panels in Fig. 3 show the methodology of the calculation: the hexagons are divided into three distinct regions (A–C), each enclosing many helical waveguides, for both the fractal and honeycomb lattices. As expected, the helicity induces a topological bandgap in the honeycomb lattice [Fig. 3a] corresponding to real-space Chern number 1, which coincides with the outcome of the natural momentum-space calculation of the Chern number (which can be used here because the honeycomb lattice is periodic). For the fractal lattice (Fig. 3b), the result of the real-space calculation is interesting, as there are many quasi-energy values having nonzero real-space Chern numbers. The most important quasi-energy range is from −0.05 to 0.05, which is within the topological bandgap of the helical honeycomb lattice where the real-space Chern number is 1. As shown in Fig. 3b, in the helical fractal lattice, the quasi-energies in the range between −0.05 and 0.05 correspond to real space Chern number 1, hence supporting the observation that edge states in this range (e.g., state numbers 95 and 93) are indeed topological. We find that the gaps between regions of eigen-values around −0.5, −0.2, 0.2 and 0.5, shaded in gray in Fig. 2b, separate different bunches of “bulk states” (states residing away from the edges) with a real-space Chern number of 0, which means that these gaps are topologically trivial.

**Fig. 4:**
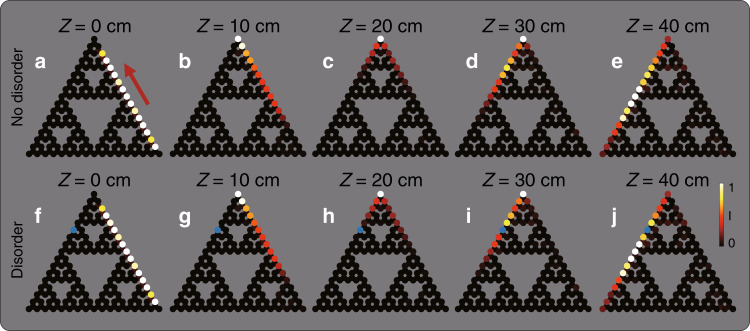
Tight-binding simulations of an edge wavepacket propagating in a fractal lattice. **a–e** Evolution of topological edge states in the fractal SG(4) lattice. **a** Intensity distribution of the initial field constructed from a truncated topological edge state in the fractal lattice. **b**–**e** Intensity distribution at propagation distances *Z* = 0, 10, 20, 30, 40cm. **f**–**j** Evolution in the fractal lattice containing an on-site disorder of $0.1c_0$, the position of which is marked by the blue dot. The wavepacket displays topologically protected edge transport around the corners and is unaffected by the disorder. The color bar indicates the field intensity. The parameters for the numerical simulation are the same as in Fig. 2

Having found unidirectional edge states with the real-space Chern number 1, we study the evolution of the edge states in evolution simulations in the presence of defects and disorder. Specifically, to verify that edge state number 95 is indeed topological, we demonstrate its ability to display topologically protected transport, the hallmark of topological physics. We launch a wavepacket at the edge of the fractal lattice and simulate its propagation (Fig. 4a–e). The initial wavepacket is a superposition of eigen edge states such that it has a finite width (see Fig. 4a). Figure 4b–e shows the light intensity at different propagation distances *Z* = 10, 20, 30, 40 cm. Clearly, the wavepacket moves along the edge of the fractal lattice and passes the corner without scattering. During propagation, the wavepacket remains confined to the edge, not penetrating into the bulk and backscattering. Next, we test the robustness to disorder. The simulation in Fig. 4f–j shows that the wavepacket can pass a defect (indicated by the blue dot in the fractal lattice)—a site with on-site disorder of strength $0.1c_0$. We find that the propagation of wavepackets of edge states in the fractal system is very robust against random on-site disorder of strength up to $0.2c_0$. The only visible difference between the initial and final wavepackets is the diffraction broadening caused by dispersion (because the edge states comprising the wavepacket evolve at slightly different rates).

The topologically protected transport of edge states in the fractal lattice is not unique to the outer edge. Supplementary Movie #1 shows a similar simulation for an inner edge in the fractal lattice (the perimeter of a hole). The excited edge state number 93 exhibits robust evolution, in the same vein as for the outer edge of the fractal lattice. Since higher-generation fractals always include more inner edges as the generation increases, we find (in simulations) that they exhibit robust propagation on the inner edges—as long as the edge includes an area that is larger than G(3)—to serve as the “bulk” region for the respective inner edge.

Altogether, we have shown that the fractal lattice of helical waveguides has a nondegenerate unidirectional edge state residing in a gap (Fig. 2), that several edge states have a real-space Chern number of 1 (Fig. 3), and that wavepackets made up of these edge states (in both the outer and inner edges) display robust transport by going around the corner and passing defects without backscattering or scattering into the bulk. ***Hence, we proved that the fractal lattice acts as a topological insulator, although there is no bulk whatsoever, and that one cannot rely on bulk-edge correspondence****.*

At this point, it is very important to emphasize that there are key differences between the fractal lattice and a helical honeycomb lattice with randomly missed sites. As we show in the Supplementary Information, Section B, a honeycomb lattice with randomly missed sites is not a topological insulator: its real-space Chern number is always in the proximity of zero, and its “edge states” do not exhibit unidirectional robust transport. It is clear that the additional symmetries of self-similarity on multiple scales, which are at the heart of fractality, are crucial for the existence of topological features in driven fractal lattices.

**Fig. 5:**
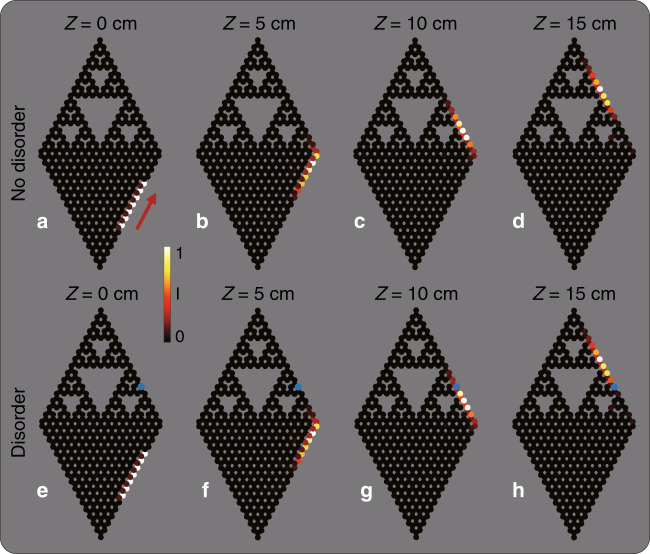
Tight-binding simulations of an edge wavepacket propagating in a hybrid lattice consisting of fractal and honeycomb lattices. The initial wavepacket (**a**, **e**) is constructed from a truncated edge state of the honeycomb lattice. **a**–**d** Propagation from the honeycomb region into the fractal region, displayed at propagation distances *Z*=0, 5, 10, 15cm. **f**–**h** Propagation into the fractal region containing a defect (blue dot) in the form of on-site disorder of $0.1c_0$. The wavepacket exhibits propagation along the edges and around the corners and bypassing disorder. The color bar indicates the field intensity. The parameters for the numerical simulations are the same as in Fig. 2

Finally, we study a hybrid lattice combining both the fractal and honeycomb lattices stitched together, as shown in Fig. 5. We launch a wavepacket comprised of topological edge states on the honeycomb side and simulate its propagation into the fractal side of the lattice. Had our lattice been strictly honeycomb, this wavepacket would propagate without scattering into the bulk and without backscattering even in the presence of disorder (or defects)—as long as the amplitude of the disorder (defect) does not close the topological gap. However, our lattice here is a hybrid: half-honeycomb, half-fractal. Hence, this numerical experiment will serve to show whether (or not) the edge states we have found support topologically protected transfer from honeycomb to fractal lattices modulated by the same helicity. The launched wavepacket shown in Fig. 5a is constructed from a superposition of edge states of the honeycomb lattice. Figure 5b–d shows the evolution, displaying the light intensity distributions at several propagation distances *Z* = 5, 10, 15 cm. The wavepacket moves along the edge of the honeycomb lattice, passes the corner without scattering, enters the fractal lattice and continues moving along the edge of the fractal lattice. Throughout propagation in the hybrid lattice, the wavepacket remains confined to the edge, does not penetrate into the bulk and does not exhibit backscattering. Moreover, the simulation in Fig. 5e–h shows that the wavepacket is able to pass a defect in the fractal lattice (its position is given by the blue dot)—a site with on-site disorder of strength $0.1c_0$. Supplementary Movies #2 and 3 show long-term propagation in this hybrid lattice, with the wavepacket encircling the lattice multiple times. Supplementary Movie #4 shows the transport with the wavepacket initially launched at the fractal lattice. Finally, Fig. S3 shows the propagation of a wavepacket in a hybrid lattice where the two components possess different nonzero real space Chern numbers. In this non-matched semi-fractal lattice, the wave partially moves along the edge and partially penetrates into the “bulk” of the fractal lattice, which indicates that this system has no topological protection. That is, for a hybrid semi-fractal system to be topological, its constituents should have the same real-space Chern number.

## Discussion

As stated earlier, our findings here are in fact a prelude to upcoming experiments in a photonic platform, which will provide experimental proof that fractal lattices can indeed behave as topological insulators. It is therefore essential to carry out wave dynamics simulations with the actual experimental parameters and examine the evolution. As shown in Supplementary Information Fig. S4, we simulate the wave dynamics of the tight-binding example of Fig. 5. Our wave dynamics simulations show good agreement with the tight-binding simulations, suggesting that what we propose here is readily experimentally accessible with the current technology.

In summary, we proposed photonic Floquet topological insulators in a fractal lattice and demonstrated robust transport along the outer and inner edges of the fractal landscape. We underpinned the difference between driven (helical) fractal lattices and lattices with randomly missed sites and showed that fractal symmetries are crucial for the existence of topological features. Finally, we showed that topological edge states can pass from the edge of the honeycomb lattice to the outer edge of the fractal structure (of the same chirality) virtually without scattering, despite the transition from two-dimensions to a fractal dimension. The parameters used in this work are all readily accessible for experiments with photonic lattices fabricated using direct laser writing^13,23^. These experiments could be the first experimental realization of topological fractal insulators^30–32^.

## Supplementary information

Supplementary Information

Supplementary Information2

Supplementary Information3

Supplementary Information4

Supplementary Information5

Supplementary Information6

Supplementary Information7
