# Supplementary material for: Photonic Floquet topological insulators in a fractal lattice
Source: Light Sci Appl. 2020 Jul 20;9:128. doi: 10.1038/s41377-020-00354-z (PMC7371641; doi:10.1038/s41377-020-00354-z)
Supplement: Supplementary file 1 — Supplementary Information [file 41377_2020_354_MOESM1_ESM.docx]

**Supplementary Information for**

**Photonic Floquet Topological Insulators in a fractal lattice**

Zhaoju Yang, Eran Lustig, Yaakov Lumer and Mordechai Segev

*Physics Department and Solid State Institute,*

*Technion– Israel Institute of Technology, Haifa 32000, Israel*

**Section A: the Sierpinski gasket** **lattice with G(5) generation**

As discussed in the main text, higher generation fractals always include more inner edges as the generation increases. In fact, the existence of inner edges in a generic property of all fractal.

We find that the modulated Sierpinski gasket (SG) of generation 5 (henceforth called G(5); shown in Fig. S1) supports topological edge states on the outer edge, and conjecture that higher generations (7, 9, etc.) always support these. We confirm (in simulations) that the topological states on the outer edge indeed display robust transport, just as G(4) does (Fig. 4 in the main text).

In addition to simulating the evolution of wavepackets on the outer edge, we also examine the propagation on the inner edges. Our simulations reveal that the inner edges of the SG fractal lattice also support robust propagation - as long as the edge is surrounded by an area that is larger than G(3), so as to serve as the “bulk” region for the respective inner edge. That is, for edge states on the inner edges to exhibit topologically protected transport – the inner edge must be well separated from other edges, by a region that is wider than the width of the edge state. For the SG lattice, we find that the inner edges must at least have a region of size G(3) separating between them and other nearby edges.

Supplementary Movies #5 and 6 present simulations showing two inner edges that support topologically protected edge states. The big blue dot indicates the initial input site.


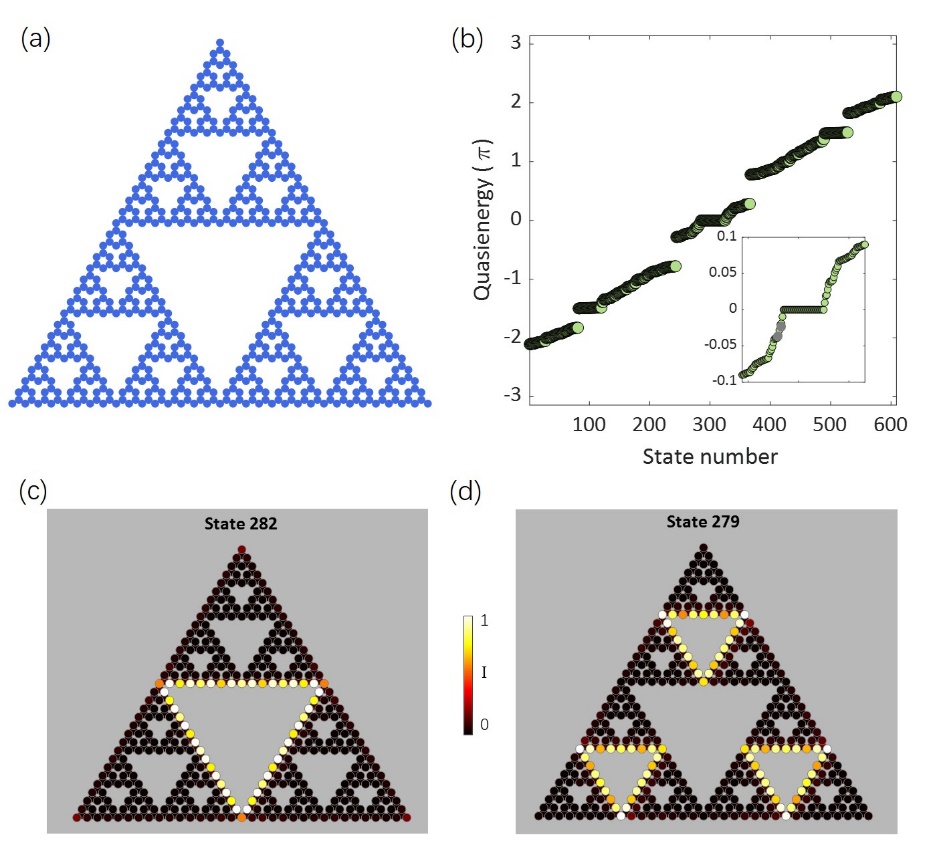


Figure S1: Inner eigen states of the G(5) fractal lattice. (a) The G(5) fractal lattice. (b) Quasienergy spectrum with $\boldsymbol{A}\left( z \right)\neq0$ for the G(5) fractal lattice. (c-d) Field intensity patterns of two eigenstates localized at internal edges of the fractal lattice (states number 279 and 282, respectively). The color bar indicates the intensity (normalized to the peak intensity in each state). The parameters for the numerical simulation are the same as in Fig. 2.

**Section B: the difference between a helical fractal lattice and a honeycomb lattice with randomly missed sites**

An instructive way to understand the role of the fractal dimensionality in the topological phase is to compare the fractal Floquet topological insulator model presented in the main text, with a Floquet topological insulator with randomly missed sites.

The main feature defining fractality is symmetry: fractal structures are made of building blocks displaying self-similarity of multiple scale. Thus, the major difference between a fractal lattice and a lattice with missed holes is that the fractal lattice has a well-defined symmetry that includes the holes, whereas a lattice with randomly missed sites only has some leftover of the original lattice. For the random case – the holes are only disruptive: they only spoil the symmetry, but never add new symmetries. In fact, this is the whole essence of fractals: symmetry over multiple generations of replicas of different sizes, which are manifested in the non-integer (fractal) dimension.

It therefore makes sense to study whether (or not) the fractal Floquet topological insulator is topologically equivalent to the Floquet topological insulator with some sites missed in the bulk. To address this issue, we simulate the honeycomb lattices with randomly missed sites. We made sure that the total number of lattice sites (and hence the number of states) in the fractal and the honeycomb with randomly missed sites are the same - to enable a fair comparison – and investigate their topological properties. [Statistically, the chance of obtaining a fractal lattice by randomly deleting nodes is negligible in a large enough lattice; hence, in the statistics, we can neglect the chance of obtaining a fractal lattice by deleting random nodes].

Figure S2 shows three examples [Panels (a-c)] of realizations of honeycomb lattices with randomly missed sites with the same number of sites as the fourth generation of the fractal honeycomb, G(4), depicted in Fig. 1 in the main text. Each of these examples displays a continuous quasi-energy spectrum.

First, we find that the real-space Chern number calculation of all the 3 cases does not indicate that these lattices are topological. For example, Panels (d-f) show the real-space Chern number calculated for cases (a-c).

Second, we check the eigenfunctions of all the states in each of the realizations, and concentrate on edge states that are “suspected” to be topological. For case (a) of the “randomly missed lattice” – there is no state that resides strictly on the edge. For cases (b, c) - the states that reside mostly on the edges (and are therefore suspected to be topological) are state #97 and state #95 [Panels (h) and (i)], respectively. For these states, we simulate the propagation and examine the robustness, as we do in Fig. 4 of the main text.

Consider first the state #97 of case (b), whose propagation is shown in the bottom row [panel (j)] of the figure, which displays the light intensity distributions at several propagation distances $Z=0, 30, 60, 90cm$. The launched wave is positioned at the blue dot, with a fixed quasi-energy identical to that of the eigen-state 97 (so as to excite only state #97). As shown in the bottom row of the figure, the excited wave moves forward and backward along the edges, penetrates into the bulk and stays localized at several positions. This means that even this most ‘suspected’ edge state is not a topological edge state. Similar behavior is observed for state #95 of case (c) (which is the most suspected to be a topological edge state for case (c) realization of the randomly missed honeycomb). From these examples, and others we have checked, we conjecture that the topological Floquet insulator with random sites missed in the bulk supports no topological edge states.

It is possible to obtain an indication for this from the real-space Chern number – which – for all “randomly missed honeycomb lattices” we checked – is always close zero, unlike the real-space Chern number of the fractal lattice –which is at the proximity of 1 (Fig 3 in the main text).

Therefore, as a result of our simulations, we conclude here that:

1. The fractal lattice can be obtained only by deleting specific nodes in the periodic lattice, not by eliminating random nodes.

2. The fractal topological insulator is not topologically equivalent to the topological Floquet insulator with random sites missed in the bulk.


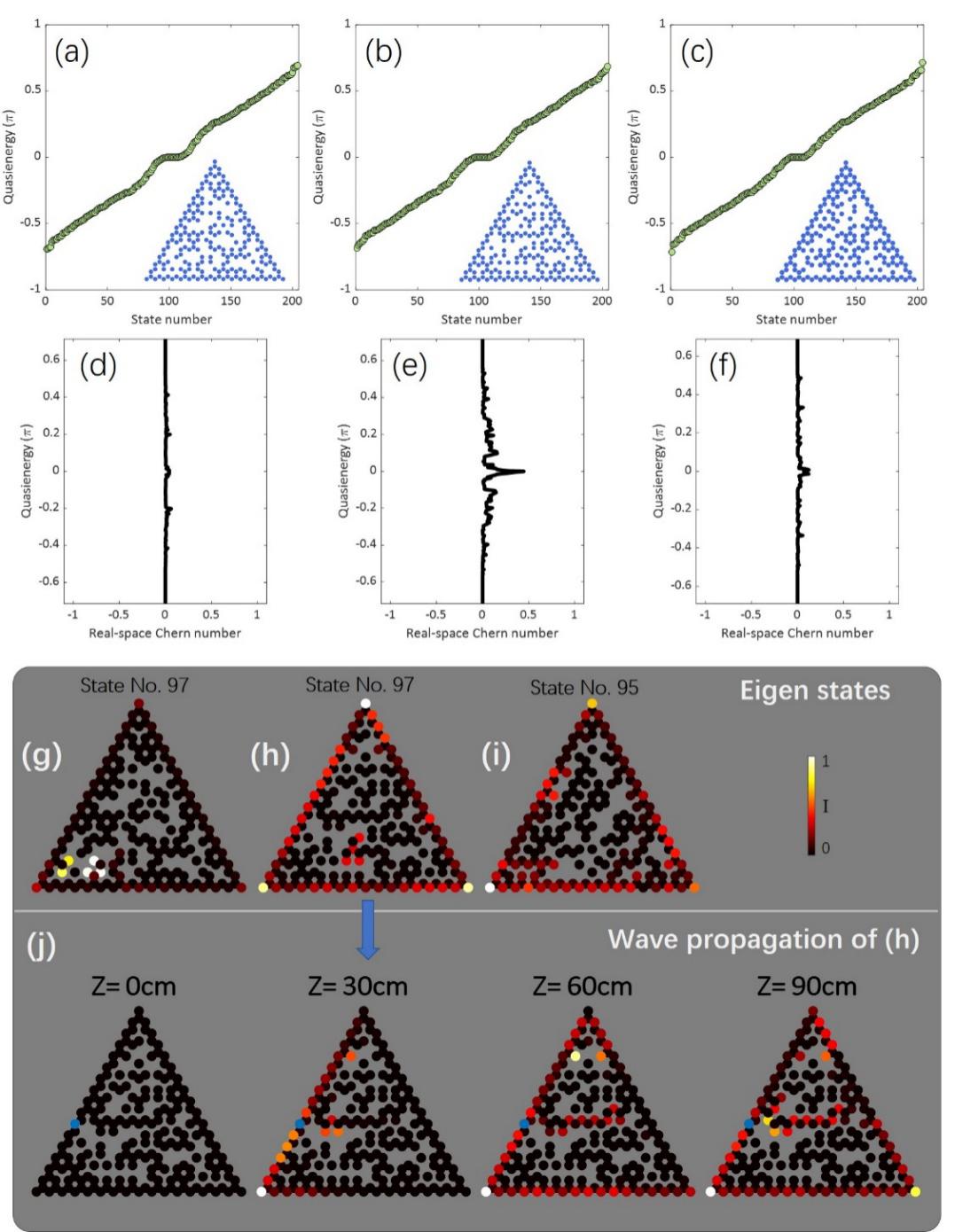


Figure S2. (a-c) Quasienergy spectrum of the honeycomb lattices with randomly missed sites. There are 204 sites in total for each case, which is the same as that of the G(4) fractal example analyzed in the main text. (d-f) Real-space Chern numbers for examples (a-c). (g-i) Middle row: three cases of eigen states. (j) Lower row: Wave propagation of ‘suspected’ edge state 97 of example (b).

**Section C: wave propagate in the hybrid system** **where the two components possess different non-zero real space Chern number**

As shown in Fig. S3, we launched a wave at the blue dot with fixed quasi-energy of $-0.066\pi$, where the half-honeycomb component possesses the real-space Chern number of 1 and the half-fractal component has the real-space Chern number of 0.3. From the simulations, we find that the wave propagates first along the edge of the honeycomb lattice, then partially keeps moving along the edge of the fractal lattice and partially penetrates into the ‘bulk’ of the fractal lattice and stays localized. The key point here is that the system with non-zero and non-integer real-space Chern number doesn’t have fully topological protection.


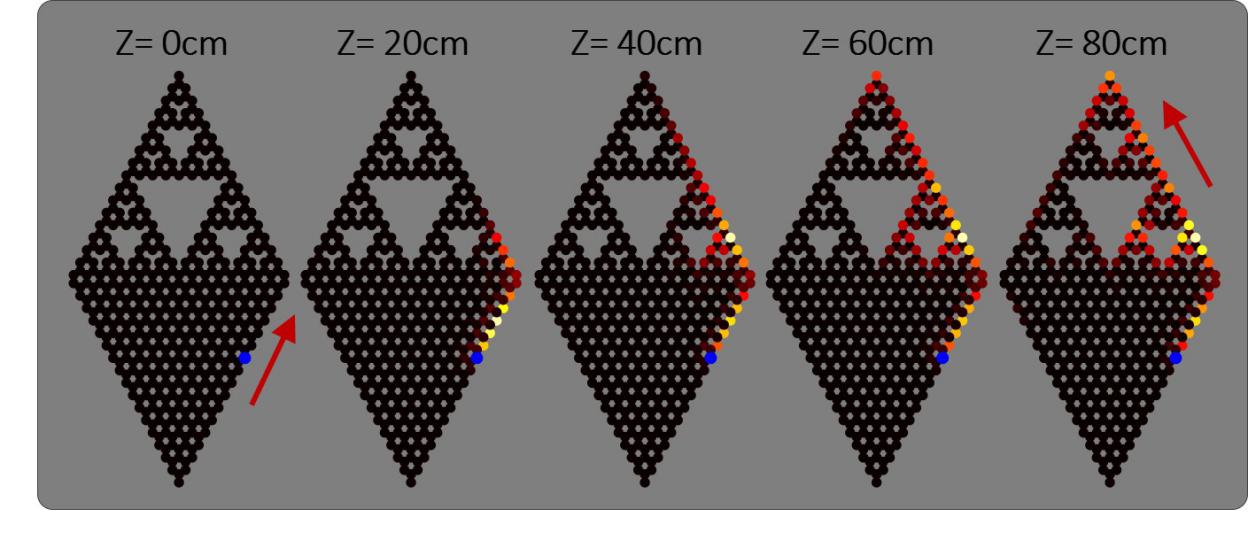


Figure S3. Wave propagation in the hybrid system, where the two components possess different non-zero real space Chern number.

**Section D: wave simulations for the hybrid lattice**

Since experiments are generally carried out in Lab space and not in Hilbert space, we carry out wave simulations to test whether the results of our tight-binding simulations hold –for some reasonable propagation distance that will allow propagation around the hybrid lattice at least once. To do that, we solve the paraxial wave equation

$i\partial_{z}\psi\left( r \right)= \frac{-1}{2k_{0}}\nabla^{2}\psi\left( r \right)- \frac{k_{0}\Delta n(r)}{n_{0}}\psi\left( r \right)$ (3)

where $\psi$ is the electric field envelope function, $k_{0}=\frac{2\pi n_{0}}{\lambda}$ is the wavenumber in the ambient medium with refractive index $n_{0}$ and $\Delta n$ is the deviation from the ambient refractive index. The parameters we used for simulations are experimentally achievable within the current technologies [22]. In these simulations, we launch a Gaussian beam at the lower-right edge of the honeycomb lattice to excite the topological edge states, as shown in Fig. S4. The field intensity at propagation distances $Z=5, 10, 15cm$ are shown in panels (a-d). The launched topological edge state moves upwards, encounters sharp corners, enters the fractal lattice and continues moving along the edge of the fractal lattice. Throughout propagation in this hybrid lattice, the wavepacket stays at the edges without any penetration into the bulk or backscattering. This topologically-protected propagation is further highlighted in the presence of local disorder of strength up to $dn=0.1\Delta n$ (while circle in (e-h)). In panels (e-h), the strength of disorder used is $dn=0.05\Delta n$. The topological behavior in the wave simulations agrees well with the results of the tight-binding simulations.


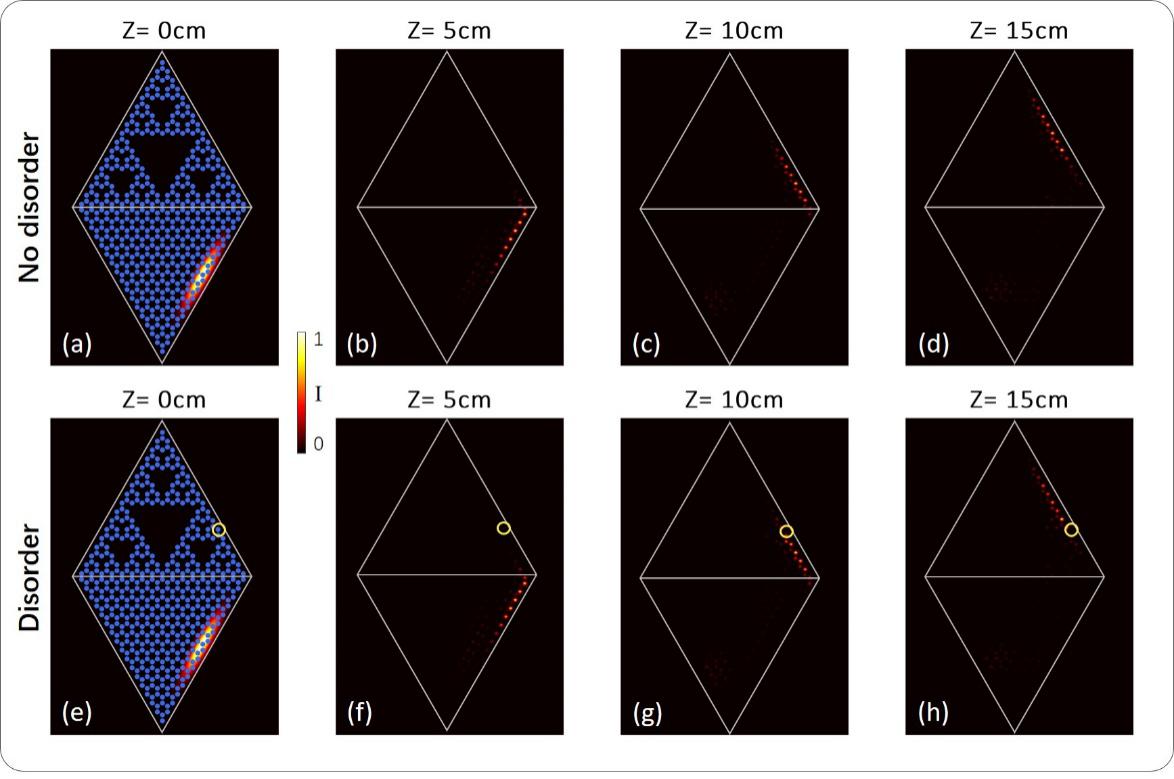


Figure S4. Beam-propagation-method (BPM) simulations of the wave packet propagating in a hybrid lattice consisting of the fractal and honeycomb lattices. The launched light field is a tilted Gaussian beam. The light distributions emerging at different propagation distances $Z=0, 5, 10, 15cm$ are presented. The wavepacket displays robust propagation without scattering away from the edge and without backscattering. (a-d). Such robust propagation also occurs in the presence of disorder (e-h). The wavepacket propagates along the edges and around the corners, and is able to bypass a defect (yellow circle) of a deviation $dn=0.05\Delta n$ from the refractive index contrast defining the waveguide at this site,$\Delta n$. The color bar indicates the field intensity. The topological protection revealed in the BPM simulations agrees well with the results in tight-binding simulations. The parameters for numerical simulations are: refractive index$n_{0}=1.45$, refractive index change $\Delta n=7.5\times{10}^{-4}$, wavelength $\lambda=0.633\mu m$, helix radius $R=10\mu m$, longitudinal frequency of the helix $\Omega=2\pi{cm}^{-1}$, lattice constant $a=14\surd3\mu m$, index change profile of a single waveguide has the form of hyper-Gaussian function $\Delta n exp(-{[{(\frac{x}{5.5})}^{2}+{(\frac{y}{1.9})}^{2}]}^{3})$.
