# Supplementary figures and images for: Photonic Floquet topological insulators in a fractal lattice
Source: Light Sci Appl. 2020 Jul 20;9:128. doi: 10.1038/s41377-020-00354-z (PMC7371641; doi:10.1038/s41377-020-00354-z)

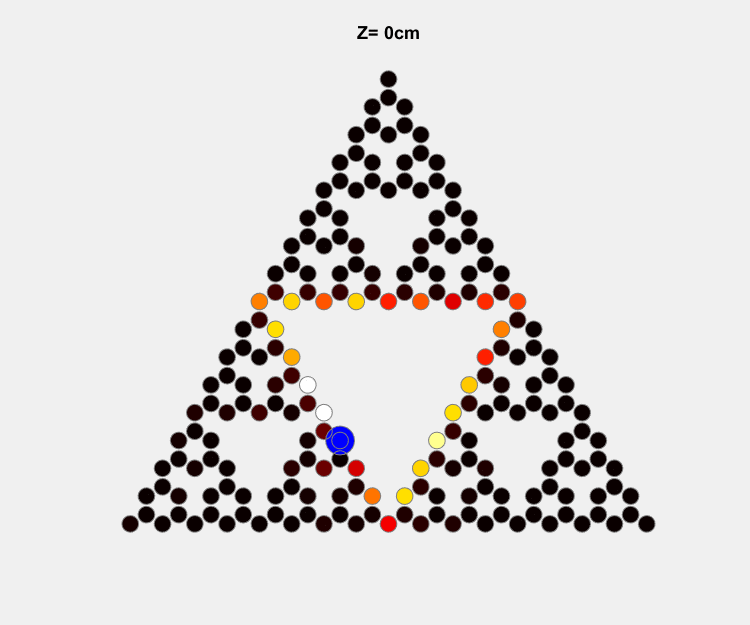

Supplement: Supplementary file 2 — Supplementary Information2 [file 41377_2020_354_MOESM2_ESM.gif]

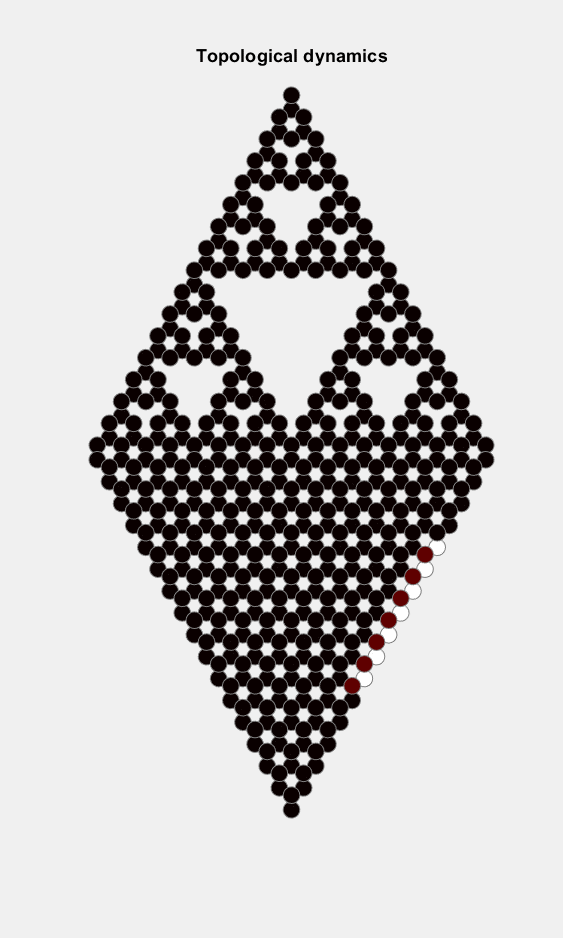

Supplement: Supplementary file 3 — Supplementary Information3 [file 41377_2020_354_MOESM3_ESM.gif]

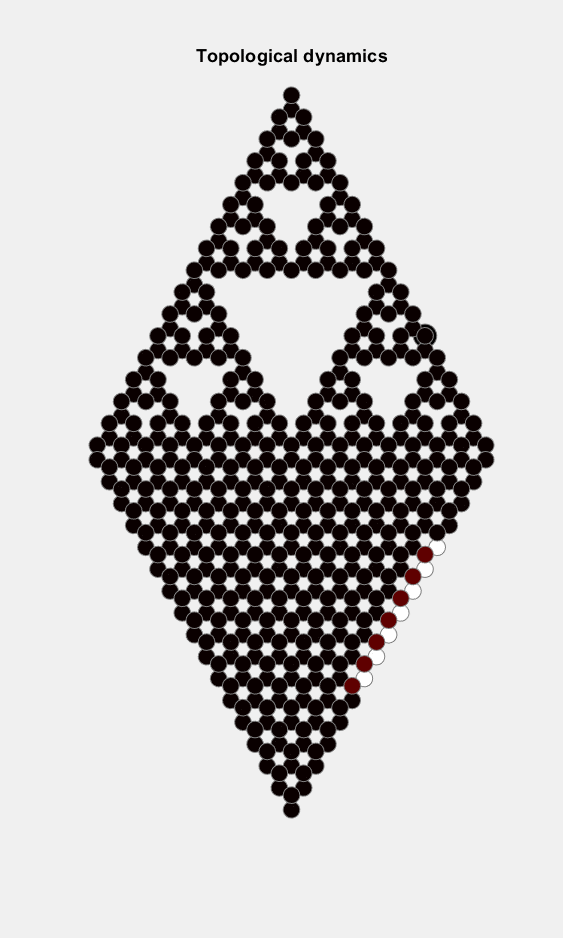

Supplement: Supplementary file 4 — Supplementary Information4 [file 41377_2020_354_MOESM4_ESM.gif]

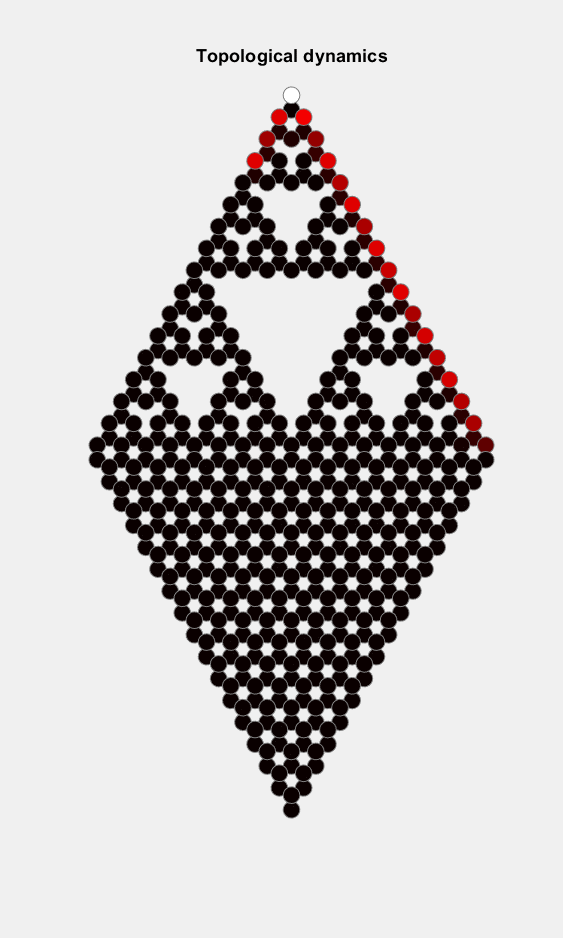

Supplement: Supplementary file 5 — Supplementary Information5 [file 41377_2020_354_MOESM5_ESM.gif]

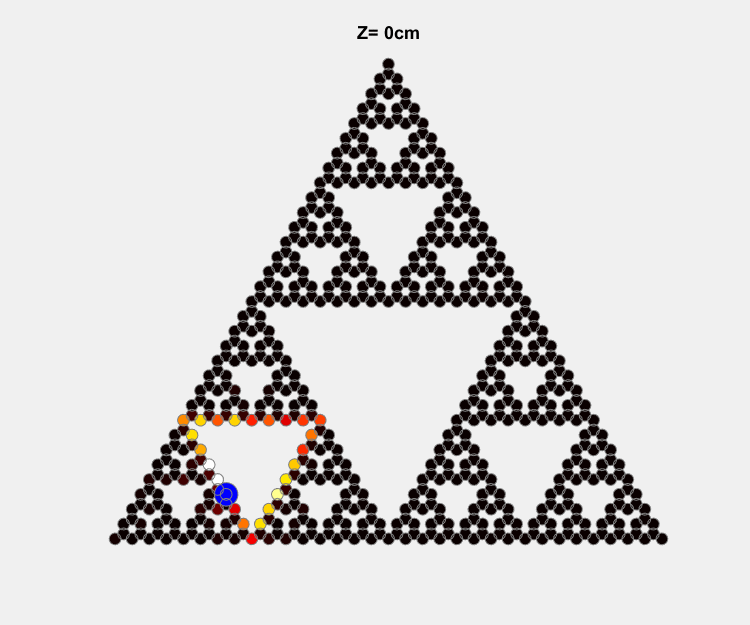

Supplement: Supplementary file 6 — Supplementary Information6 [file 41377_2020_354_MOESM6_ESM.gif]

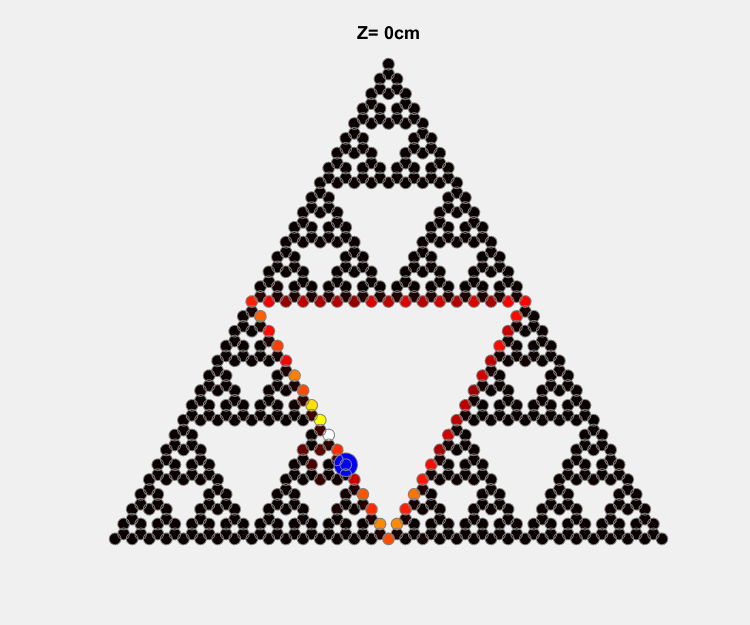

Supplement: Supplementary file 7 — Supplementary Information7 [file 41377_2020_354_MOESM7_ESM.gif]
